# Transmembrane Parkinson’s disease mutation of PINK1 leads to altered mitochondrial anchoring

**DOI:** 10.1016/j.jbc.2025.108253

**Published:** 2025-02-03

**Authors:** Raelynn Brassard, Elena Arutyunova, Emmanuella Takyi, L. Michel Espinoza-Fonseca, Howard S. Young, Nicolas Touret, M. Joanne Lemieux

**Affiliations:** 1 Department of Biochemistry, University of Alberta, Edmonton, Alberta, Canada; 2 Li Ka Shing Institute of Virology, University of Alberta, Edmonton, Alberta, Canada; 3 Neuroscience and Mental Health Institute, University of Alberta, Edmonton, Alberta, Canada; 4 Department of Internal Medicine, Center for Arrhythmia Research, University of Michigan, Ann Arbor, Michigan, USA

**Keywords:** MD simulation, Parkinson’s disease, PARL, proteostasis, rhomboid protease

## Abstract

Parkinson’s disease is a devastating neurodegenerative disease resulting from the death of dopaminergic neurons in the substantia nigra pars compacta of the midbrain. Familial and sporadic forms of the disease have been linked to mitochondrial dysfunction. Pathology has been identified with mutations in the *PARK6* gene encoding PTEN-induced kinase 1 (PINK1), a quality control protein in the mitochondria. Disease-associated mutations at the transmembrane (TM) region of PINK1 protein were predicted to disrupt the cleavage of the TM region by the PARL (presenilin-associated rhomboid-like) protease at the inner mitochondrial membrane. Here, using microscopy, kinetic analysis, and molecular dynamics simulations, we analyzed three Parkinson’s disease–associated TM mutations; PINK1-C92F, PINK1-R98W, and PINK1-I111S, and found that mitochondrial localization and cleavage by the PARL protease were not significantly impaired. However, clearance of hydrolyzed PINK1-R98W appears to be compromised because of altered positioning of the protein in the outer mitochondrial membrane, preventing association with translocase of the outer membrane complexes and slowing cleavage by PARL. This single amino acid change slows degradation of proteolyzed PINK1, increasing its accumulation at the outer mitochondrial membrane and resulting in increased mitophagy and decreased mitochondrial content among these cells.

Mitochondrial dynamics and surveillance mechanisms are crucial for maintaining homeostasis of the mitochondrial network (1). As such, mitochondrial dysfunction has been linked to various illnesses and diseases, such as migraines, multiple sclerosis, and Parkinson’s disease (PD) (2, 3, 4, 5). PD is the second most common neurodegenerative disease characterized by motor symptoms as the result of a loss of dopaminergic neurons in the substantia nigra pars compacta of the midbrain (6). These dopaminergic neurons are particularly susceptible to mitochondrial dysfunction and damage because of various factors including little to no myelination, decreased mitochondrial mass, intrinsically low energy production, and intrinsic pacemaker activity (7, 8). Through fusion and fission, mitochondria can share organellar contents, undergo biogenesis, as well as segregate damaged portions of a mitochondrion for removal (9). The dynamic nature of these organelles and the many layers of regulation allow them to maintain the metabolic requirements of the cell, providing energy in the form of ATP.(9, 10)

One such mechanism to maintain the integrity of the mitochondrial network is through the selective degradation of damaged mitochondria in a process known as mitophagy, a specialized form of autophagy (11, 12, 13). This critical process is mediated through the kinase PINK1 (PTEN-induced kinase 1) and E3 ubiquitin ligase, Parkin (11, 14, 15). In a healthy mitochondrion, the transmembrane (TM) helix of PINK1 is inserted into the inner mitochondrial membrane (IMM) *via* the translocase of the outer membrane (TOM) and translocase of the inner membrane (TIM) complexes (16, 17, 18). Here, the mitochondrial targeting sequence (MTS) is removed *via* mitochondrial processing peptidases in the matrix. Furthermore, the TM segment of PINK1 is cleaved by a resident protease in the IMM, presenilin-associated rhomboid-like (PARL) protease, and subsequently released to the cytosol where it is further degraded by the 26S proteosome (19, 20, 21). However, upon mitochondrial damage and depolarization, the import of PINK1 to the IMM is impaired. Consequently, the full-length (FL) protein (FL-PINK1) begins to accumulate on the outer mitochondrial membrane (OMM), where the protein will dimerize and undergo autophosphorylation and in addition phosphorylate ubiquitin moieties of OMM proteins and Parkin, and the formation of polyubiquitin chains leads to the initiation of mitophagy (22, 23). As such, PINK1 plays a critical role in mitochondrial quality control. This is a highly regulated process, and mutations in genes encoding both PINK1 and Parkin have been associated with familial forms of young-onset Parkinson’s disease (YOPD) as well as other human diseases (24, 25, 26, 27, 28, 29). Proteolytic processing of PINK1 by PARL is critical in its ability to provide its gatekeeping role in the mitochondria. Mutations that impair mitophagy through the PINK1–Parkin pathway would result in an aberrant accumulation of damaged mitochondria that would disrupt the integrity of the mitochondrial network. The opposite may be true if PINK1–Parkin-mediated mitophagy is overactive, resulting in clearance of otherwise healthy mitochondria, and depletion of the network, which may lead to cell death.

To date, therapies for PD rely heavily on addressing the symptoms (6, 30). However, no current therapies are curative or halt the progression of the disease; this is largely because of a lack of understanding in disease etiology. As the importance of mitochondrial integrity begins to become more understood in the field, a variety of mitochondrial enhancers are now in clinical trials with hopes of slowing disease progression. One such method is through PINK1 enhancers to increase the basal level of mitophagy as a means of maintaining the integrity of the network in those who have deficient mitophagy levels (6, 8, 31).

Nearly 100 mutations in PINK1 have been identified in YOPD patients across all domains of the protein (32, 33); the amino-terminal MTS, single-pass TM segment, cytosolic kinase domain, and C-terminal domain. Patients with these mutations in PINK1 experience a young-onset form of the disease, with onset occurring as young as 32 years old. Due to their proximity to the cleavage site by PARL, TM mutations of PINK1 have been predicted to interfere with PARL cleavage (14). PARL is an intramembrane rhomboid protease that resides in the IMM and is known to play a role in mitochondrial proteostasis through the cleavage of various TM substrates (34, 35, 36, 37, 38). PARL preferentially cleaves PINK1 under healthy cellular conditions; however, upon mitochondrial depolarization, PARL switches to predominately cleaving a mitochondrial phosphatase PGAM5 (39, 40). Compound point mutations in the *Pink1* gene were identified in a 37-year-old PD patient, resulting in C92F, R464H.(41, 42) Neuronal SH-SY5Y cells expressing C92F revealed abnormal aggregation of mitochondria (14). Previous reports demonstrated that C92F mutation did not impair dimerization of PINK1 (43); therefore, further analysis is required to determine the influence of this variant of protein trafficking and proteolysis to uncover its role in disease pathogenesis. A mutation of Arg98, which resides below the PARL cleavage site at Ala103, to a tryptophan was observed in an Italian family with YOPD.(44) Reports claim that in these patients, the manifestation began in the lower limbs and exhibited a slower disease progression (44). The substitution of a positively charged residue to a bulky hydrophobic residue is likely to change conformational changes in the helix and therefore plausible to predict that this mutant may impede PARL-mediated cleavage of the PINK1-TM. Cellular studies have displayed an accumulation of FL-PINK1 in cells expressing this R98W variant (45), suggesting this variant may have a gain-of function influence; however, it has not been shown if this influence is due to protein trafficking and import into the IMM or due to disruption in PARL-mediated proteolysis.

Finally, a mutation proximal to the intermembrane space interface, downstream of PARL cleavage site, Ile111Ser, was found to be pathogenic (44). The substitution of a nonpolar to small polar residue could cause significant structural changes and destabilize the helical structure of the TM. This suggests that the protein may result in misfolding, which could influence proteolysis in the membrane. Cellular assays have demonstrated that the mutation does not increase Parkin recruitment (13), but slight accumulation of PINK1 is observed (45) therefore, further studies are required to determine the mechanism of pathogenesis of this mutation.

Here, we studied the processing of the aforementioned patient-identified mutations surrounding the TM of PINK1-C92F, R98W, and I111S (44) and demonstrate PARL-mediated cleavage of the TM remains intact for the PINK1 variants assessed. Cellular localization studies however indicate some mutations cause the retention of PINK1 in the OMM. Molecular dynamics (MD) simulations rationalize the retention in the mitochondrial membranes, providing a rationale for the PD phenotype.

## Results

### Mutations in PINK1-TM region do not influence PINK1-mCherry localization to the mitochondria in HeLa cells

**Figure 1:**
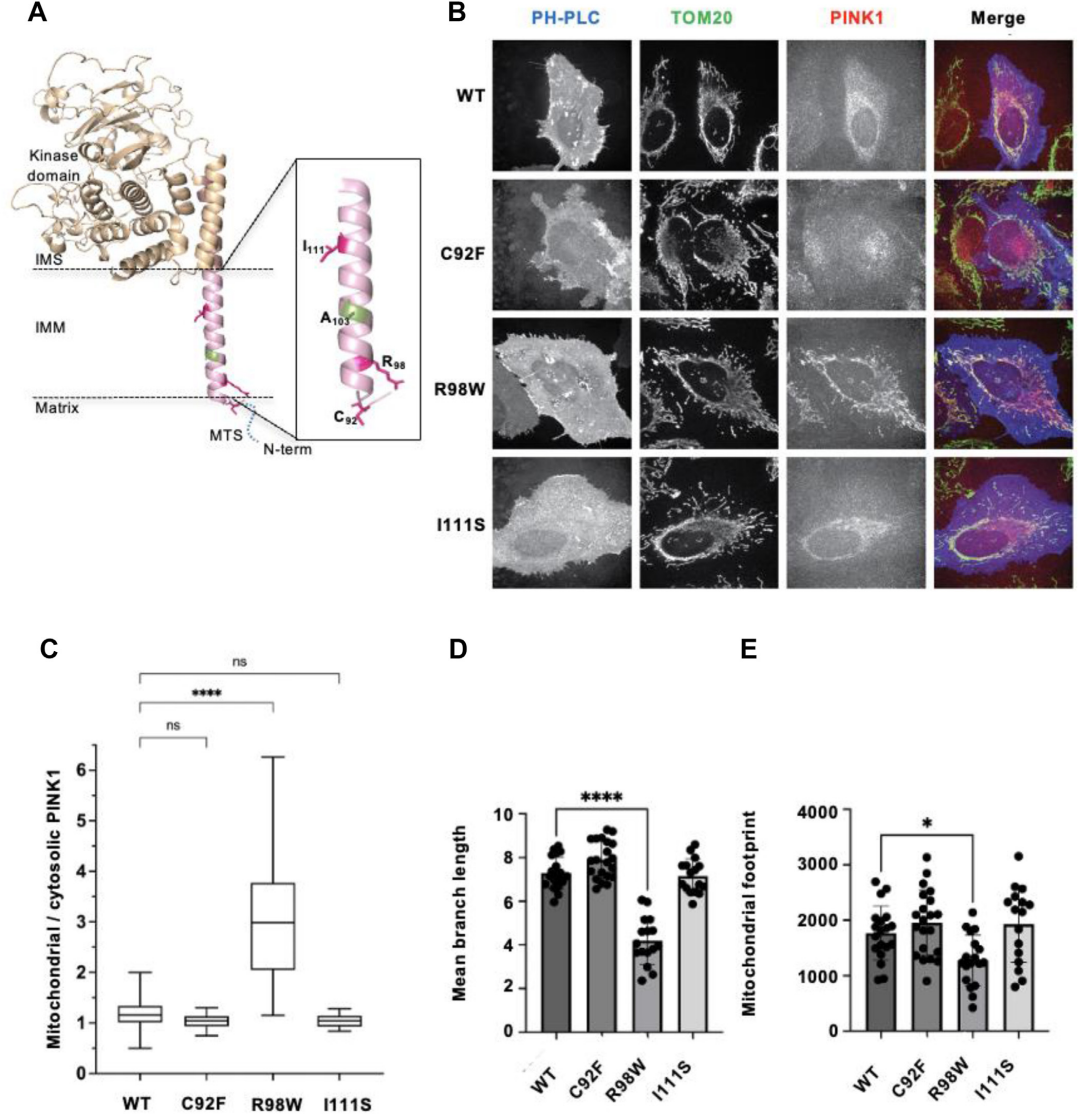
**Mitochondrial accumulation of PINK1 variants associated with PD.***A*, topological diagram depicting PINK1 variants found in the transmembrane region near the P1 position of PARL cleavage site at Ala103: C92F, R98W, and I111S. *B*, representative confocal fluorescence microscopy images (maximum intensity projection of z-stacks) of HeLa cells cotransfected with mCherry-tagged PINK1-WT or PD variant PINK1 and the iRFP-PH-PLC and immunostained for TOM20. Composite figure displaying merge of PINK1-mCherry (*red*), TOM20 (*green*), and cell boundaries (*blue*). *C*, quantification of PINK1-mCherry fraction in mitochondria *versus* cytosol from *A*. The *central line* marks the median, whereas the edges of the *boxes* depict the 25th and 75th percentiles. The *whiskers* extend to extreme data points. Data are from ∼20 cells imaged in three independent experiments. *p* Values obtained using one-way ANOVA with Brown–Forsythe test. *D* and *E*, quantification of mitochondrial morphology; mean branch length (*D*) and mitochondrial footprint (*E*), using MiNa (ImageJ). Experiments were replicated with a minimum of n = 3. Data are represented as mean ± SEM (one-way ANOVA with Brown–Forsythe test, ∗*p* < 0.05; ∗∗∗*p* < 0.0005, ns denotes no significance). PARL, presenilin-associated rhomboid-like protease; PD, Parkinson’s disease; PINK1, PTEN-induced kinase 1; TOM20, translocase of the outer membrane 20.

Three PD-associated mutations are located within the proposed TM region of PINK1 (residues 89–112): C92F, R98W, and I111S (Fig. 1*A*). Due to the proximity of these mutations to the cleavage site by PARL at A103, they may lead to misfolding and improper mitochondrial targeting of PINK1 or defects in the cleavage or recognition of PINK1 by the PARL protease. Defects in the processing of these mutants would lead to an imbalance of mitophagy and mitochondrial damage in these cells, which could contribute to the neuronal cell death observed in PD. Previous findings report incomplete processing of an R98F variant of PINK1 (13).

To determine the mechanism behind the PD phenotype, we assessed whether these mutations in PINK1 influence its trafficking in HeLa cells. PINK1 was cloned with an mCherry tag at the C terminus for confocal imaging and immunoblotting (Fig. 1*B*). Previously, PINK1 has been reported to be turned over too rapidly to be detected *via* confocal microscopy; therefore, to ensure selection of PINK1-transfected cells, iRFP-PH domain of phospholipase C (PLC), which binds PIP2, was cotransfected at a ratio of 1:5 with PINK1-mCherry. This allowed us to identify transfected cells expressing exogenous PINK1 as well as delineate the cell border for signal integration. The localization of PINK1-mCherry signal was correlated with the mitochondrial network using antibodies against a known OMM marker, TOM20. The relative fraction of PINK1 and mutants on mitochondria was quantified by measuring the ratio of mCherry intensity (for PINK1) within the mitochondria (defined using TOM20) to the mCherry intensity within the cytosol outside mitochondria. Cells expressing PINK1-WT have an approximate 1:1 ratio of mitochondrial to cellular PINK1 localization (Fig. 1*C*), as it is rapidly cleaved and removed from the mitochondria under normal cellular conditions. The cells expressing the PINK1-C92F and PINK1-I111S PD variants display a similar cellular distribution of PINK1 as cells expressing PINK1-WT. In contrast, the cells expressing PINK1-R98W display significant mitochondrial retention, with 2.98 times more PINK1 localized to the mitochondria compared with the cytosol (Fig. 1, *B* and *C*). While these data suggest that all assessed PD variants are successfully localized to the mitochondria in HeLa cells, the accumulation in the mitochondria seen with the PINK1-R98W implies this variant may have altered processing.

In addition, cells expressing PINK1-R98W have fragmented mitochondria when compared with PINK1-WT (Fig. 1*D*), similar to what is observed with cells treated with carbonyl cyanide m-chlorophenyl hydrazone (CCCP), an uncoupler of mitochondrial potential (Fig. S2, *A* and *B*). Quantification of the mitochondrial network using the Fiji ImageJ plugin MiNA showed significant reduction in the summed branch length of the mitochondria and decreased mitochondrial volume in cells expressing PINK1-R98W compared with PINK1-WT, whereas no significant change was seen with other PD variants analyzed (Fig. 1*D*). Decreased mitochondrial mass along with mitochondrial accumulation of PINK1-R98W suggests that this mutation may have an overall decreased mitochondrial network when compared with cells expressing PINK1-WT.

### Accumulation of FL and cleaved forms of PINK1-R98W are observed in cellular assays

#### PINK1-R98W does not show cleavage defect across various cell lines

**Figure 2:**
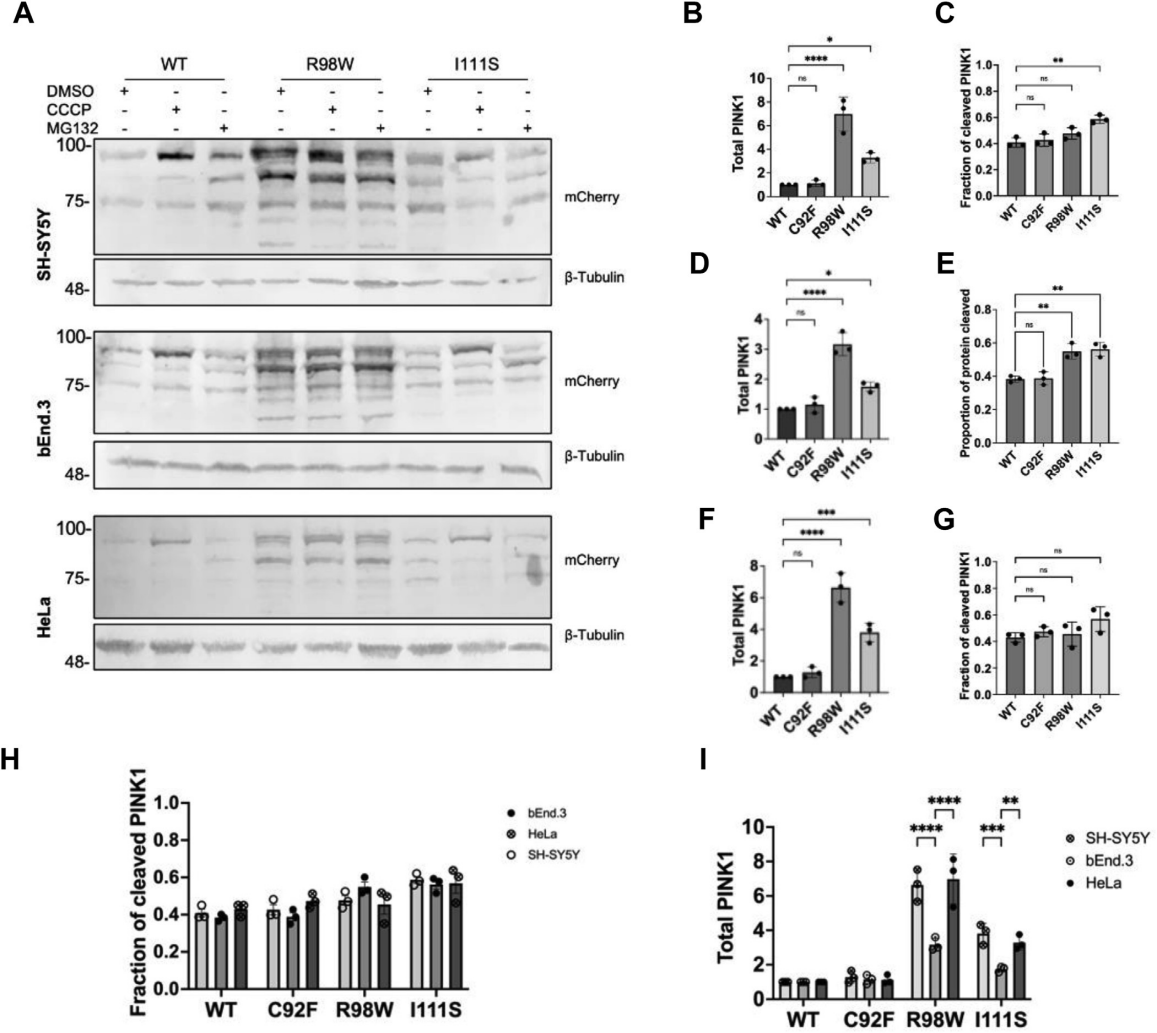
**PINK1-R98W accumulation observed across various cell lines**. *A*, SH-SY5Y, b.End.3, or HeLa cells transfected with PINK1-mCherry WT or PD variant were treated for 6 h with DMSO (control), CCCP (10 μM), or MG132 (10 μM). Whole-cell lysate proteins were separated by SDS-PAGE and immunoblotted with anti-mCherry antibody to assess PINK1 processing. IB:antitubulin is a loading control. *B*–*G*, total PINK1 and PINK1 fragment distributions of PD variants compared with PINK1-WT. Quantification was performed by densitometry analysis and normalized to the amount of PINK-WT being set to 1. *H* and *I*, comparison of PINK1 cleavage (*H*, no significance between any cell type) and abundance (*I*) between SH-SY5Y, bEnd.3, and HeLa cells. Data are from n = 4 and represented as mean ± SEM (two-way ANOVA with Šidák multiple comparison test, ∗*p* < 0.05; ∗∗∗*p* < 0.0005, ns denotes no significance). CCCP, carbonyl cyanide m-chlorophenyl hydrazine; DMSO, dimethyl sulfoxide; PD, Parkinson’s disease; PINK1, PTEN-induced kinase 1.

To assess the cleavage of PINK1-WT and PD variants, we examined whole-cell lysates of HeLa, neuronal SH-SY5Y, and brain endothelial bEnd.3 cells transfected with WT or PD variants of PINK1-mCherry. It was crucial to assess the processing of the protein in various cell lines and cell types, as previous studies of PINK1 cleavage performed in varying cell lines displayed significantly different processing (10, 11). Cells were treated with either dimethyl sulfoxide (DMSO) (control), CCCP, a disruptor of mitochondrial electrochemical, or MG132, an inhibitor of the proteasome, lysed, and proteins from whole-cell lysate were separated *via* SDS-PAGE and immunoblotted using anti-mCherry antibody to avoid detection of endogenous PINK1. Analysis of PINK1 processing between the various cell lines revealed that PINK1-WT along with C92F displays low levels of all PINK1 fragments because of rapid turnover of the protein (Fig. 2). Following uncoupling with CCCP, we observe an accumulation of FL-PINK1-WT, C92F, and I111S, which is to be expected because of diminished import to the IMM that is known to be dependent on mitochondrial membrane potential. Conversely, with the PINK1-R98W variant, while cleavage of the protein is observed, the overall abundance of the protein is significantly higher in all assessed cell lines (Fig. 2). No difference is noted between the cleavage pattern on PINK1-WT and C92F and I111S in the presence of DMSO, CCCP, or MG132, suggesting these variants are properly processed by the PARL protease. However, as noted previously, both PINK1-R98W FL and cleaved forms of the protein appear to be accumulating.

### Kinetic analysis of proteolysis shows the R98W mutation in PINK1-TM region does not harbor a cleavage defect

**Figure 3:**
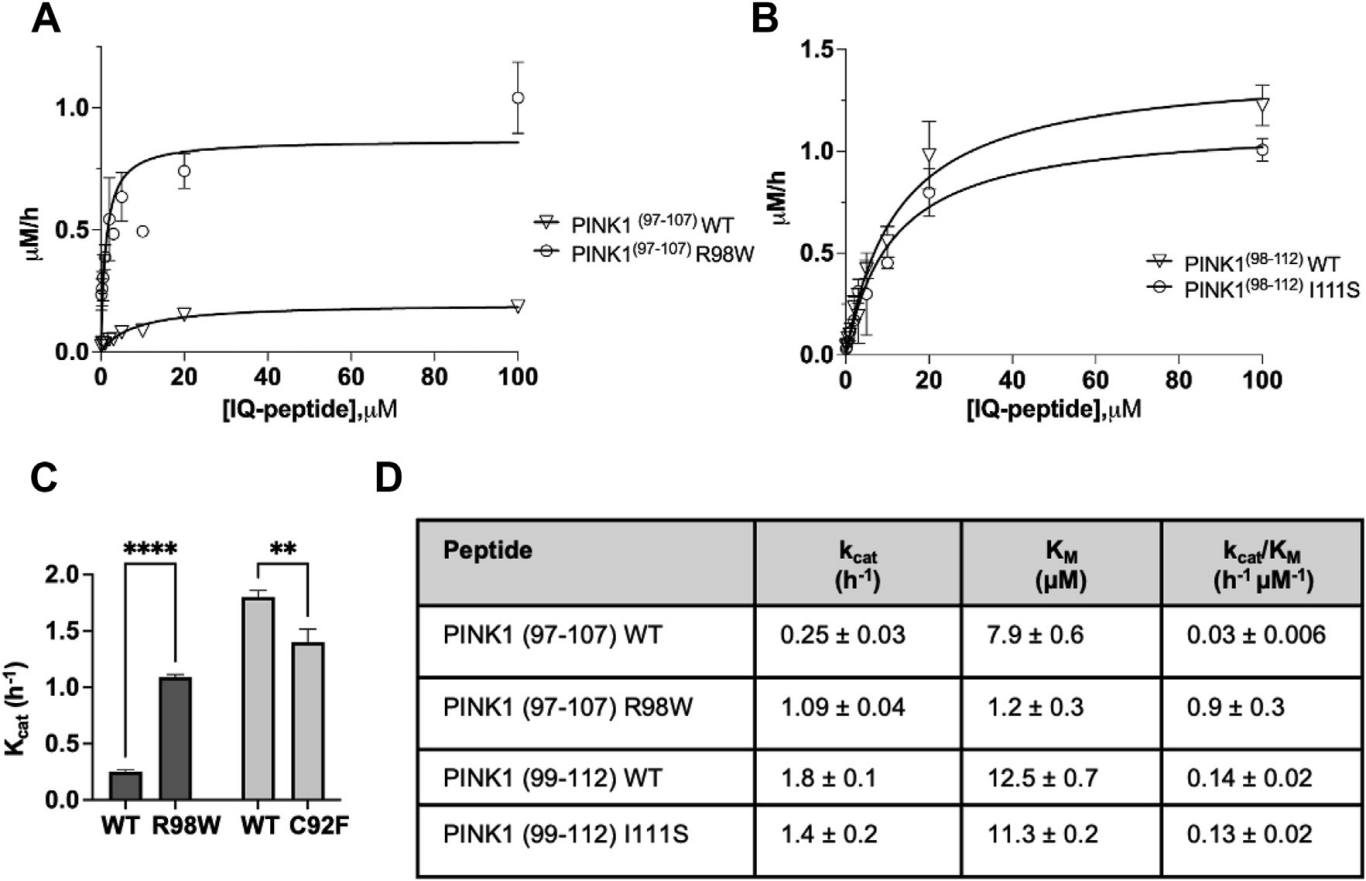
**Catalytic parameters obtained for cleavage of PD-associated PINK1 variants**. *A*, representative Michaelis–Menten kinetic curves for IQ-PINK1(97–107)-WT and IQ-PINK1(97–107)-R98W cleaved by PARLNK1. *B*, representative Michaelis–Menten kinetic curves for IQ-PINK1(98–112)-WT and IQ-PINK1(98–112)-R98W cleaved by PARLNK1. *C*, *k*cat of IQ-PINK1(97–107)-WT and IQPINK1(97–107)-R98W and IQ-PINK1(98–112)-WT and IQ-PINK1(98–112)-I111S. *D*, table of kinetic parameters determined for the above peptides. Paired *t* tests were performed with a cutoff of *p* < 0.05 to indicate a statistically significant difference. Experiments were conducted in duplicate with an N = 3. Data are represented as mean ± SEM (∗*p* < 0.05; ∗∗∗*p* < 0.0005). IQ, internally quenched; PD, Parkinson’s disease; PINK1, PTEN-induced kinase 1.

In order to assess whether these PINK PD mutations led to alterations in cleavage rate by PARL protease, we designed internally quenched (IQ) peptidic substrates based on the sequence of PINK TM region to test the kinetic parameters using *in vitro* assay. The mature form of human PARL, HsPARLΔ77, was cloned in pPICZA vector, expressed in GS115 *Pichia pastoris* expression system, and purified following previously described protocol (36, 46). Using PINK1 peptides harboring the mutation sites IQ-PINK1^97–107^-R98W, or IQ-PINK1^99–112^-I111S, and their respective WT peptides, IQ-PINK1^97–107^-WT and IQ-PINK1^99–112^-WT (Fig. S1), we determined the catalytic parameters of their HsPARLΔ77-mediated cleavage. We observe no defect in turnover rate of IQ-PINK1^97–107^-R98W by HsPARLΔ77 compared with the corresponding IQ-PINK1^97–107^-WT peptide (Fig. 3, *A* and *C*, *D*). In fact, the cleavage of the IQ-PINK1^97–107^-R98W peptide had a higher *k*_cat_ and a 30-fold increase in catalytic efficiency than its matched WT peptide. This reinforced the *in vivo* data (Fig. 2) showing that PINK1-R98W does not have a cleavage defect as previously suggested (18). Furthermore, the cleavage of IQ-PINK1^99–112^-I111S was diminished compared with that of IQ-PINK1^99-112^-WT (Fig. 3, *B* and *C*, *D*). Due to issues with peptide solubility, we were unable to assess PINK1-C92F in this *in vitro* assay.

The absence of an apparent cleavage defect taken together with the mitochondrial retention of PINK1-R98W, and cleavage observed in cell lysates, suggests that the PINK1-R98W results in a processing defect independent of cleavage by PARL.

### R98W expression in SH-SY5Y increases level of mitophagy

**Figure 4:**
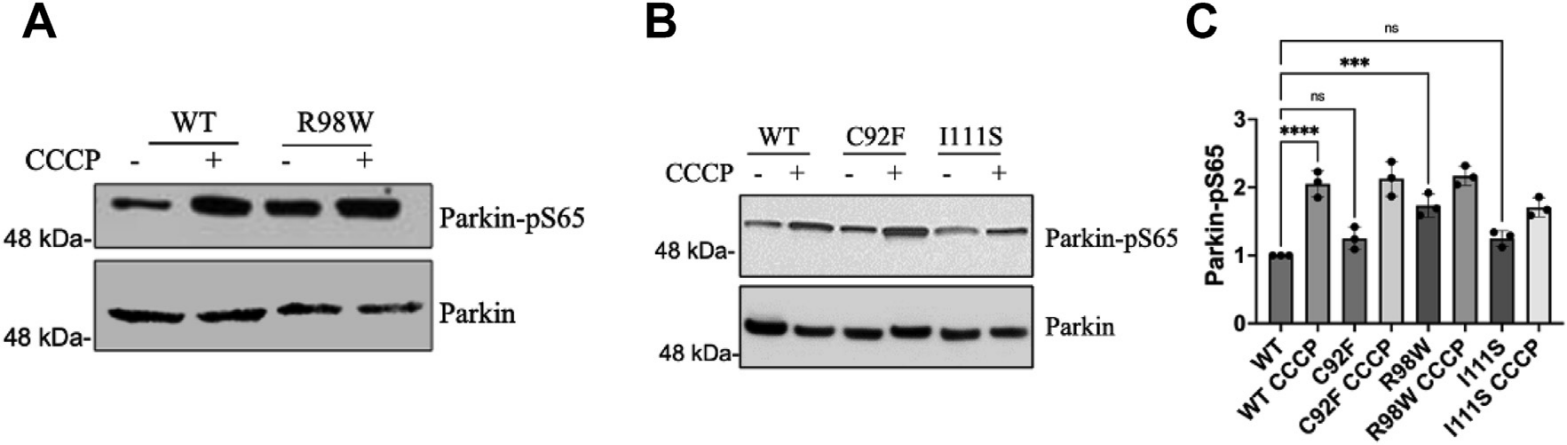
**TM variants cause alteration in mitochondrial function.***A* and *B*, immunoblot of SHSY5Y whole-cell lysates with phospho-Parkin and Parkin antibodies treated with either DMSO (control) or CCCP (10 μM) for 3 h. *C*, comparison of the amount of Parkin-p65s with different TM variants expressed. Quantification of Parkin-p65s immunoblot was performed by densitometry analysis and normalized so the density of PINK1-WT band was set to 1. N = 3 and represented as mean ± SEM (one-way ANOVA with Brown–Forsythe test, ∗*p* < 0.05; ∗∗∗*p* < 0.0005, ns denotes no significance). CCCP, carbonyl cyanide m-chlorophenyl hydrazine; DMSO, dimethyl sulfoxide; TM, transmembrane.

In neuronal cell line, SH-SY5Y cells, but not HeLa cells, endogenous expression of Parkin allows PINK1-mediated mitophagy to proceed in damaged or mutation-bearing cells. When PINK1 is trapped on the OMM because of disruption of electrochemical potential, such as when cells are treated with CCCP, the kinase domain initiates phosphorylation of cytosolic and OMM proteins, including Parkin at Ser65 (47). Increased phosphorylation at Parkin-Ser65, using a phospho-Parkin-Ser65-specific antibody, was observed in an immunoblot of SH-SY5Y cells transiently transfected with PINK1-R98W-mCherry (Fig. 4). This increase in pS65-Parkin could be mimicked by treatment of WT-PINK1 cells with CCCP (Fig. 4).

### PINK1-R98W has altered positioning in the mitochondrial membrane

**Figure 5:**
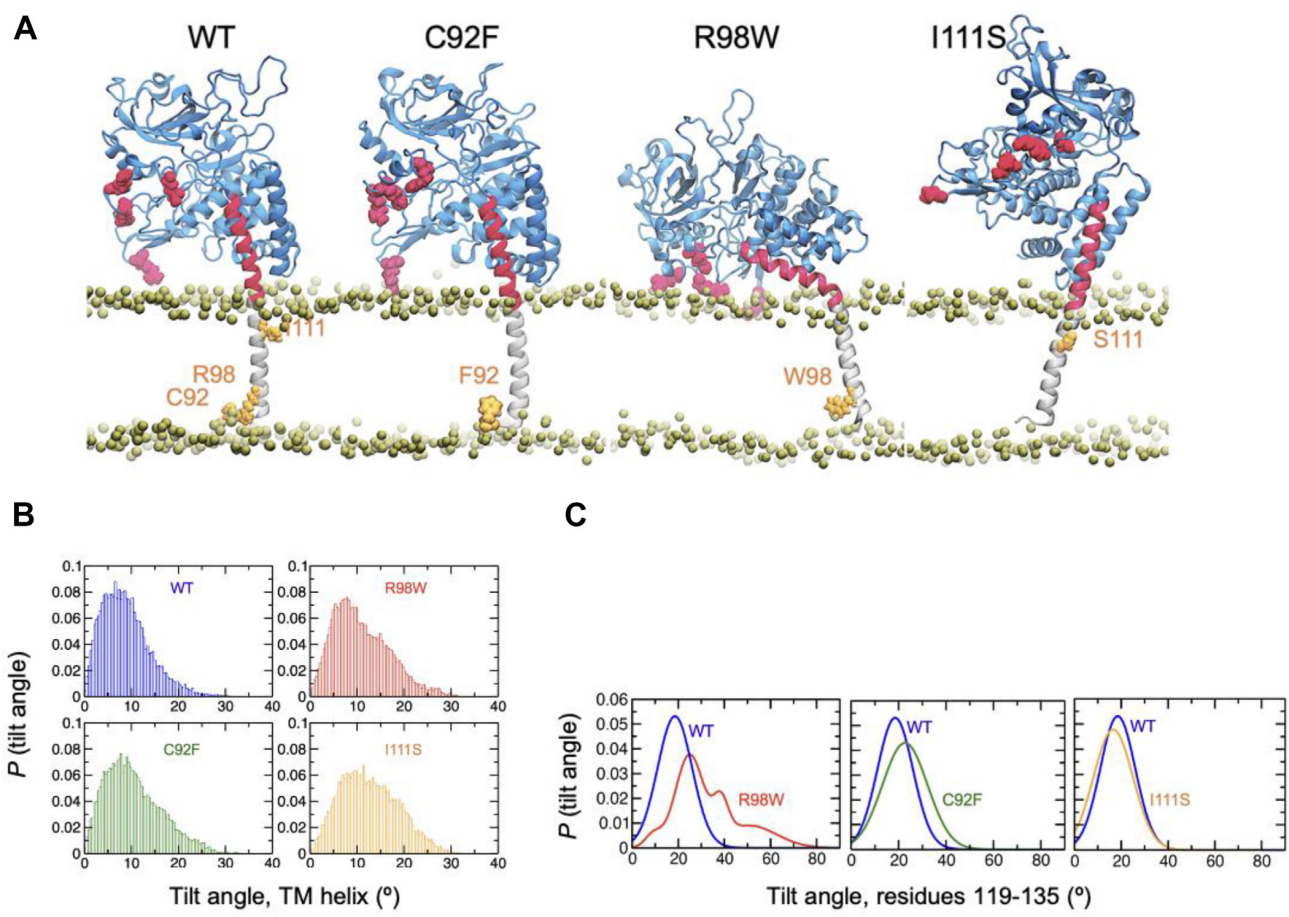
**TM variants cause alteration in mitochondrial membrane insertion.** Molecular dynamic simulations of PINK1-WT, PINK1-C92F, PINK1-R98W, and PINK1-I111S in the outer mitochondrial membrane. *A*, AlphaFold model was used to predict TM boundaries and protein structure. Model depicting tilt of TM and soluble domains of PINK1 in the presence of absence of PD mutants. *B*, analysis of tilt angle of the PINK1TM helix (residues 89–118) as determined by MD simulations *C*, tilt angle of soluble helix (residues 119–135) of PINK1 variants compared with WT PINK as determined by MD simulations. MD, molecular dynamics; PINK1, PTEN-induced kinase 1; TM, transmembrane.

To shed light on the effect the mutations might have on the positioning of TM region within the lipid bilayer, we performed MD simulations on PINK1-WT and PD variants using the AlphaFold-predicted boundaries of the TM region of PINK1, C92-A118 (Fig. 5). In PINK1-WT, Arg98 appears to perform a snorkeling function that allows Arg119, Arg120 to position close to the phospholipid bilayer headgroups. However, this function is abolished when Arg98 is mutated to Trp. This single point mutation induces a slight tilt in the TM helix (Fig. 5*B*), resulting in a significant kink in the extended soluble helical region of the protein (Fig. 5*C*), exposing Arg119,120 to solvent environment. This causes the soluble kinase domain to be positioned closer to the OMM when compared with PINK1-WT (Fig. 5). This tilted soluble domain is not observed in simulations of PINK1-C92F or I111S (Fig. 5*C*), suggesting this is a unique feature upon mutation of residue R98. The altered positioning in the OMM as well as the change observed in the soluble domain likely influences the ability of PINK1-R98W TM helix to be inserted into the IMM *via* TOM20. Recent work suggests that while PINK1-R98W interacts with TIM23, it is not pulled down by TOM20 (48). This would be supported by the MD simulations showing altered placement in the OMM. As such, the import of PINK1-R98W is likely quite slow, relying solely on the electrochemical potential rather than import complexes to reach PARL in the IMM. In addition, we predict that the increased interaction of the soluble domain with the mitochondrial membrane prevents cleaved PINK1 to be released to the cytosol after cleavage by PARL, contributing to mitochondrial retention of PINK1 and subsequent shift in mitophagy balance, leading to dysfunction.

## Discussion

PINK1, along with Parkin, plays a critical role in the selective removal of damaged or unhealthy mitochondria from the cell *via* the process known as mitophagy, while preserving the healthy mitochondria. Upon loss of the electrochemical potential (Δψm), PINK1 becomes trapped on the OMM where it recruits Parkin to initiate mitophagy. Under healthy mitochondrial conditions, PINK1 is imported to the IMM, where it is cleaved by the resident rhomboid protease PARL, and the newly truncated PINK1 returns to the cytosol, before being degraded by the proteosome. For this reason, despite containing an MTS at its amino terminus, PINK1 is present in both cytosolic and mitochondrial compartments of the cell and rapidly turned over, indicative of healthy mitochondria. Cellular studies provided initial evidence suggesting that impaired PINK1 processing because of mutations within the TM region can lead to mitochondrial dysfunction (13, 14).

Here, we characterized three PD-PINK1 variants that are found in patients with familial forms of YOPD, specifically PINK1-C92F, R98W, and I111S. An examination of mCherry-labeled PINK1 variants in HeLa cells with confocal image analysis demonstrated R98W significantly accumulates in the mitochondria compared with PINK1-WT, which is present in both cytosolic and mitochondrial compartments of the cell. However, in all assessed cell lines, PINK1-R98W is partially processed to the 52 kDa fragment, with a similar ratio of PINK1 cleavage to PINK1-WT, suggesting that processing is not inhibited by this mutation. Furthermore, upon examination of catalytic parameters of cleavage of the IQ-PINK1-R98W peptide *in vitro*, no significant defect in cleavage was noted compared with IQ-PINK1-WT peptide, suggesting that the mutation of Arg98 to Trp98 does neither affect the recognition nor the cleavage of PINK1-R98W by PARL. Previous cellular studies also emphasized a severe defect in PINK1-R98W processing, suggesting that PARL-mediated cleavage of the variant was impaired (13, 18, 45). However, our *in vitro* kinetic results and immunoblotting suggest that PINK1-R98W is indeed efficiently cleaved by PARL-mediated proteolysis. Interestingly, the R98W mutation resulted in a marked increase in the catalytic efficiency of PARL-mediated cleavage of the IQ-PINK1 peptide by 30-fold. This suggests that W98 enhances the interaction between the PINK1 substrate and PARL, potentially facilitating the cleavage process. This unexpected increase in catalytic efficiency indicates that the R98W mutation may alter substrate dynamics or binding affinity in a way that improves enzymatic activity, rather than disrupting it.

**Figure 6:**
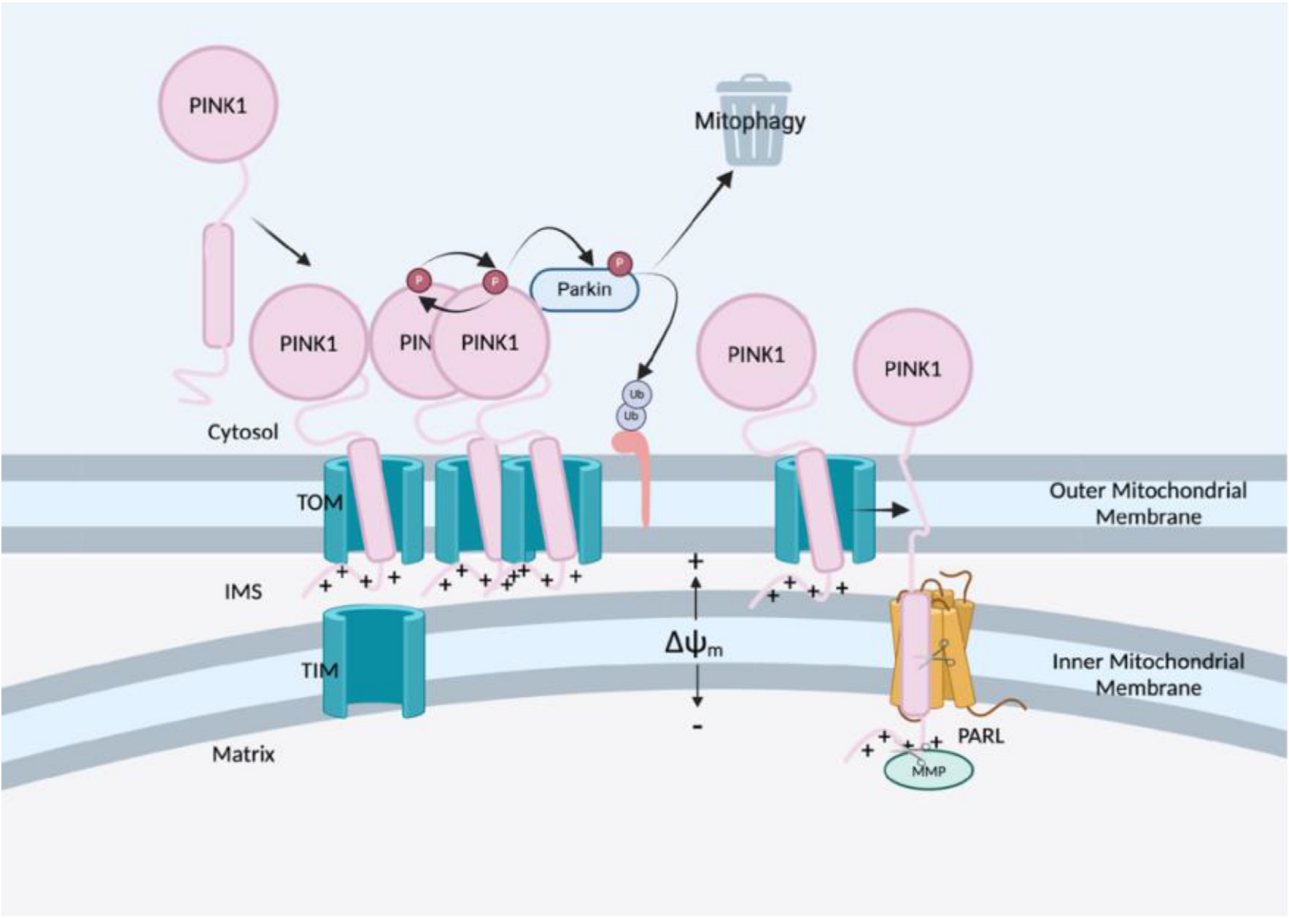
**Mutations in PINK1 transmembrane region can influence either trafficking to the IMM or the binding to and cleavage by to the intramembrane protease PARL, inducing a recruitment of Parkin and signaling for mitophagy in otherwise healthy mitochondrion.** Our data indicate that PINK1-R98W is cleaved by PARL in the IMM, but because of changes in membrane positioning, has increased interaction with the membrane that hinders subsequent release and degradation, leading to mitochondrial accumulation. IMM, inner mitochondrial membrane; PARL, presenilin-associated rhomboid-like protease; PINK1, PTEN-induced kinase 1.

Therefore, we show that despite successful cleavage by PARL, PINK1-R98W is unable to be released to the cytosol to be degraded by the proteosome (Fig. 6) and speculate this retention is due to other factors. TM helices containing arginine are thermodynamically stable when buried in the core of the lipid bilayer (49). Intermolecular interaction analyses revealed burial of centrally located arginine *via* multiple mechanisms: local bilayer distortion, guanidium snorkeling, and peptide shifting along the bilayer (50). The mutation of an arginine residue to a hydrophobic tryptophan in the lipid membrane, results in helix distortion in relation to the lipid bilayer and potential negative downstream effects in its ability to be correctly trafficked to the IMM. In addition, the apparent cleavage by PARL accompanied by mitochondrial accumulation of this cleavage product, rather than its usual fate of cytosolic degradation, suggests that retrograde transport into the cytoplasm is defective postcleavage, likely resulting in clogging of outer membrane import machinery and OMM accumulation.

The retention and accumulation of the mutated PINK1 protein in the OMM leads to unnecessary mitophagy of otherwise healthy mitochondria. This imbalance in the mitochondrial damage and mitophagy pathway likely leads to cell death in the substantia nigra pars compacta, which is particularly susceptible to mitochondrial dysfunction, contributing to PD pathogenesis. Increased mitophagy in cells expressing PINK1-R98W was confirmed by increased phosphorylation of Parkin at S65. For mutation R98W, our results support a loss of spatial positioning in the OMM and retention. Arginine in TM residues is known to play key roles in positioning in lipid bilayer, snorkeling to the hydrophobic core, and associating with charged lipid headgroups (49, 51, 52). A loss of the central Arg98 removes the key residues positioning it in the middle of the bilayer hydrophobic core. With a charged to hydrophobic residue, mutation R98W may also assist with retention in the hydrophobic bilayer.

This observation suggests that the R98W mutation fundamentally alters the structural and functional dynamics of PINK1 at the mitochondrial membranes, with profound implications for its activity. In PINK1-WT, Arg98's snorkeling function positions critical residues like Arg119 and Arg120 close to the phospholipid headgroups, stabilizing the protein within the lipid bilayer and ensuring proper orientation and import. The R98W mutation disrupts this stabilization, creating a tilt in the TM region and a kink in the helical region. This not only exposes Arg119 and Arg120 to the solvent environment but also brings the soluble kinase domain closer to the OMM, impacting its interactions with import machinery.

The structural shift likely affects PINK1's ability to engage with the TOM20–TIM23 import complexes. While PINK1-R98W retains some interaction with TIM23, the MD simulations suggest that the tilted orientation of the TM helix prevents efficient insertion into the IMM through TOM20. This may explain why PINK1-R98W import is slower and primarily dependent on the electrochemical gradient, bypassing the typical reliance on import complexes.

In addition, the simulations predict that the altered placement of PINK1-R98W on the OMM increases interactions between its soluble domain and the mitochondrial membrane. This could hinder the release of cleaved PINK1 to the cytosol after PARL-mediated cleavage in the IMM. In PINK1-WT, cleaved fragments are typically released to the cytosol to participate in mitophagy signaling. The inability of PINK1-R98W to release its cleaved products may significantly impair the recruitment of downstream effectors like Parkin, disrupting the mitophagy pathway and leading to an accumulation of damaged mitochondria.

Taken together, these findings highlight a cascade of structural and functional disruptions caused by the R98W mutation. The mutation's unique impact on the TM helix orientation, soluble domain positioning, and interactions with mitochondrial membranes underpins its pathological effects, offering a potential explanation for the impaired mitophagy observed in cells expressing PINK1-R98W. Future studies could focus on validating these predictions experimentally, such as through *in vitro* reconstitution of import dynamics, analysis of cleaved fragment release, and real-time tracking of mitophagy processes in cells.

The mutation of isoleucine 111 to a serine results in increased total PINK1 levels in all assessed cell lines to varying degrees. Previous cellular studies report impaired cleavage of PINK1 I111S.(13, 45) However, while we report increased total levels of PINK1 in I111S variant, the ratio of cleaved:uncleaved protein is not significantly different to that of WT-PINK1, and in addition, we do not observe a cleavage defect in our *in vitro* kinetic analysis. This point mutation decreases the overall hydrophobicity of the TM helix (53); therefore, it is possible that its trafficking to the IMM is slowed compared with that of WT-PINK1. This is consistent with the appropriate cytoplasmic:mitochondrial ratio of PINK1-I111S observed in our cellular studies.

The C92F variant did not show significantly different distribution of proteolytic fragments or mitochondrial retention of the mutant protein compared with PINK1-WT, suggesting they do not have any processing defects. This leaves the remaining question: how do these mutations cause PD if they appear to be correctly processed and proteolyzed by the PARL protease? It is possible that the mutation destabilizes the protein structure. Deas *et al*. (14)reported that cells expressing PINK1-C92F displayed abnormal distribution and aggregation of their mitochondria; however, this result is not replicated in our cellular studies.

Our research helps us rationalize the mechanism behind the PD phenotypes associated with familial mutations located in the juxta membrane or TM region of PINK1. While none of these mutations directly impede PARL-mediated proteolysis, each variant assessed impart different molecular dysfunction of the PINK1 protein.

## Experimental procedures

### Cell lines

HeLa cells (human epithelial cervix, adenocarcinoma, American Type Culture Collection [ATCC] CCL-2) and bEnd.3 cells (mouse brain endothelial cells, ATCC CRL-2299) were grown in high-glucose Dulbecco’s modified Eagle’s medium and supplemented with 10% fetal bovine serum (Wisent, Inc). SH-SY5Y cells (ATCC CRL-2266) were grown in Eagle’s minimum essential media (ATTC 30-2003) supplemented with 10% fetal bovine serum and 1% penicillin–streptomycin. All cells were maintained in a 5% CO_2_ incubator at 37 °C.

### Immunolabeling and confocal microscopy

HeLa cells seeded on glass coverslips were transiently transfected with 0.25 μg pmCherry-C1-PINK1 and 0.05 μg of iRFP-PH-PLC delta1 (gift from Pietro De Camilli, Addgene plasmid #66841; http://n2t.net2/addgene:66,841; Research Resource Identifier: Addgene_66841) (54) using Fugene6 transfection reagent (Promega Corporation) at ∼50% confluency. Three hours prior to fixation, the cells were incubated with 10 μM of CCCP, MG132, or vehicle control DMSO. Twenty-four hours post transfection, cells were fixed with 3.7% paraformaldehyde and 0.2% glutaraldehyde for 12 min at room temperature, reduced in 0.2% sodium borohydride in PBS, then blocked and permeabilized in PBS containing 3% bovine serum albumin and 0.1% Triton for 30 min at 21^°^C. Blocked coverslips were incubated with 1:500 diluted primary antibody (Rabbit anti-TOM20, sc-11415; Santa Cruz Biotechnologies) for 30 min at 21^°^C and then washed five times with PBS containing 0.1% Triton X-100. Coverslips were incubated in diluted fluorescent secondary antibody (donkey anti-rabbit Alexa Fluor 568; Thermo Fisher Scientific) for 30 min at room temperature for 30 min, protected from light, and washed three times with PBS containing 0.1% Triton in PBS for 10 min each. Cells were postfixed in 4% paraformaldehyde, and then mounted onto glass slides using ProLong Gold Antifade Reagent (Thermo Fisher Scientific). Images were collected using a 60x/1.42 oil objective lens (Olympus; now Evident) on an Olympus IX-81 motorized microscope equipped with a Yokogawa CSUX1 spinning disk confocal scan head (Quorum Technologies). Images were acquired using a Hamamatsu EMCCD (C9100-13) interfaced in Volocity software (Quorum Technologies), and imaging parameters were kept constant for each channel across all experiments. The samples were illuminated with 491, 561, and 692 nm pumped diode laser wavelength. Z-stacks were recorded using 0.25 μm step size and are displayed as maximum intensity projections. Image quantification was performed using MATLAB and ImageJ.

### Image analysis and mitochondria enrichment measurement

Quantification of the relative amount of PINK1 variants present in mitochondria was determined using MATLAB (MathWorks). The intensity of all channels was corrected for nonuniform background using a wide Gaussian (standard deviation of 10 pixels) followed by background subtraction. The cell area for each transfected cell was determined using Otsu segmentation in the membrane probe channel (iRFP-PH-PLCdelta1) (55). The mitochondria were segmented using Otsu thresholding method on the TOM20 channel. The enrichment of PINK1 in mitochondria was measured by dividing the mean PINK1 channel intensity in the mitochondria mask by the mean PINK1 channel intensity in the cell outside the mitochondria mask. Therefore, a ratio >1 represents a mitochondrial enrichment of PINK1, and a ratio <1 represents a cytosolic enrichment of PINK1. Images with incorrect cell or mitochondrial mask segmentation were removed from the analysis. Merging of fluorescent channels was performed using ImageJ.

### Western blot analysis

HeLa, SH-SY5Y, or bEnd.3 cells grown to ∼50% confluency in 6-well plates were transiently transfected with 1.5 μg DNA and 4.5 μl of Fugene6 (Promega Corporation). Cells were incubated 6 h with transfection mixture and then returned to growth media for an additional 18 h. Six hours prior to lysis, cells were incubated with 10 μM CCCP, (carbobenzoxy-l-leucyl-l-leucyl-l-leucinal), MG132, or vehicle control DMSO. Whole-cell lysates were generated by washing cells with PBS 24 h post-transfection, and then cells were detached with prewarmed Accutase (Gibco) for 10 min at 37^°^C. Cells were pelleted at 5000*g* at 4^°^C for 5 min and washed with cold PBS and then lysed in cold radioimmunoprecipitation assay buffer containing cOmplete mini, EDTA-free protease inhibitor cocktail (Roche) on an ice bath for 20 min. Lysates were cleared by centrifugation at 15,000*g* for 25 min at 4^°^C. The resulting supernatant was separated on a 4 to 14% SDS-PAGE. Proteins were electrotransferred to methanol-activated polyvinylidene fluoride membranes (0.45 μm; Millipore) and blocked overnight at 4^°^C in Tris-buffered saline with 0.1% Tween-20 and 5% bovine serum albumin. Membranes were incubated with Rabbit anti-mCherry (1:2500 dilution) (Rockland) and mouse anti-αTubulin (1:4000 dilution) (Rockland) for 1 h with shaking at 21^°^C and then washed (3 × 5min) in Tris-buffered saline with 0.1% Tween-20. Blots were then incubated with Donkey anti-Rabbit 680 (1:4000 dilution) and Donkey anti-Mouse 800 (1:4000 dilution) fluorescent secondary antibodies. Fluorescent signals were detected using the Li-Cor Odyssey Fc imaging system and image studio acquisition software. Data quantification was performed using Fiji ImageJ (http://fiji.sc/). Data shown are representative of three independent experiments.

### Expression and purification of recombinant human PARLΔ77

PARLΔ77 was expressed and purified as previously described (36).

### IQ-based protease kinetic assay

Assays with IQ-PINK1 variants were conducted as previously described (56). For EDANS/Dabcyl 10mer IQ-peptides, 1 mg lyophilized peptide (Biobasic) was dissolved in 1 ml DMSO to obtain stock concentrations. The peptide was incubated with buffer (50 mM Tris–HCl [pH 7.0], 150 mM NaCl, 10% glycerol, and 0.1% *[[*-dodecyl-d-maltoside in a 364-well black-bottomed plate at 37°C for 30 min in a multiwell plate reader (Biotek Cytation 5 cell imaging multimode reader; Agilent). Following preincubation, PARLΔ77 was added to a final concentration of 0.8 μM to initiate the cleavage reaction and reaching a final reaction volume of 100 μl. For IQ peptide assays, the DMSO concentration was kept constant at 5%. The concentration of substrate ranged from 0.1 to 100 μM. Fluorescence readings were taken every 3 min over a 2.5-h time course (ƛ_ex_ = 414 nm and ƛ_em_ = 530 nm). The initial velocity was determined from the relative fluorescence unit over time; background fluorescence for each substrate concentration over the time course was subtracted. Relative fluorescence units were converted to concentration (micromolar) by determining the slope of the maximum change in fluorescence observed for each substrate concentration when fully digested by proteinase K. GraphPad Prism software (GraphPad Software, Inc) was used for Michaelis–Menten analysis. Minimum of three experimental replicates with technical duplicates were used for data analysis.

### MD simulations

We used an atomic model of human PINK1 from the AlphaFold Protein Structure Database (57, 58, 59) (entry: Q9BXM7) to simulate the dynamics of WT and mutants C92F, R98W, and I111S. In this study, we simulated residues C92 through L581 as the structure of this sequence is predicted with good confidence, according to the AlphaFold model. The proteins were inserted into a 100 × 100 Å bilayer of 1-palmitoyl-2-oleoyl-*sn*-glycero-3-phosphocholine lipids. The protein–lipid systems were solvated using TIP3P water molecules with a minimum margin of 20 Å in the *z*-axis between the edges of the periodic box and the protein, and Na^+^ and Cl^-^ ions were added to neutralize the system and to produce an NaCl concentration of ∼150 mM. Preparation of the systems was done using the CHARMM-GUI web interface (60). We performed molecular simulations with AMBER20 on Tesla V100 GPUs (61) using the AMBER ff19SB force field (62). We maintained a temperature of 310 K with a Langevin thermostat and a pressure of 1.0 bar with the Monte Carlo barostat. We used the SHAKE algorithm to constrain all bonds involving hydrogens and allowed a time step of 2 fs. We first performed 5000 steps of steepest-descent energy minimization followed by equilibration using two 25-ps MD simulations using a canonical ensemble (NVT), one 25-ps MD simulation using an isothermal–isobaric ensemble (NPT), and two 500-ps MD simulations using the NPT ensemble. The equilibrated systems were used as a starting point to perform three independent 1-μs replicates of each system.

## Data availability

All data are contained within the article.

## Supporting information

This article contains supporting information.

## Conflict of interest

The authors declare that they have no conflicts of interest with the contents of this article.
