# Supplementary material for: Transmembrane Parkinson’s disease mutation of PINK1 leads to altered mitochondrial anchoring
Source: J Biol Chem. 2025 Feb 3;301(3):108253. doi: 10.1016/j.jbc.2025.108253 (PMC11910106; doi:10.1016/j.jbc.2025.108253)
Supplement: Supporting Information [file mmc1.docx]

**Supporting Information**

| **Peptide name** | **Peptide construct** |
| --- | --- |
|  |  |
| IQ-PINK1^(99-112)^ - WT | (DABCYL)-AVFLAFGLGLGLIE-(EDANS) |
| IQ-PINK1^(99-112)^ - I111S | (DABCYL)-AVFLAFGLGLGLSE-(EDANS) |
| IQ-PINK1^(97-107)^ - WT | Arg-Lys-(DABCYL)-GRAVFLAFGLG-Glu-(EDANS)-Arg |
| IQ-PINK1^(97-107)^ - R98W | Arg-Lys-(DABCYL)-GWAVFLAFGLG-Glu-(EDANS)-Arg |
|  |  |

**Supplemental Fig 1.** Table of peptides used in this study and their respective sequences.


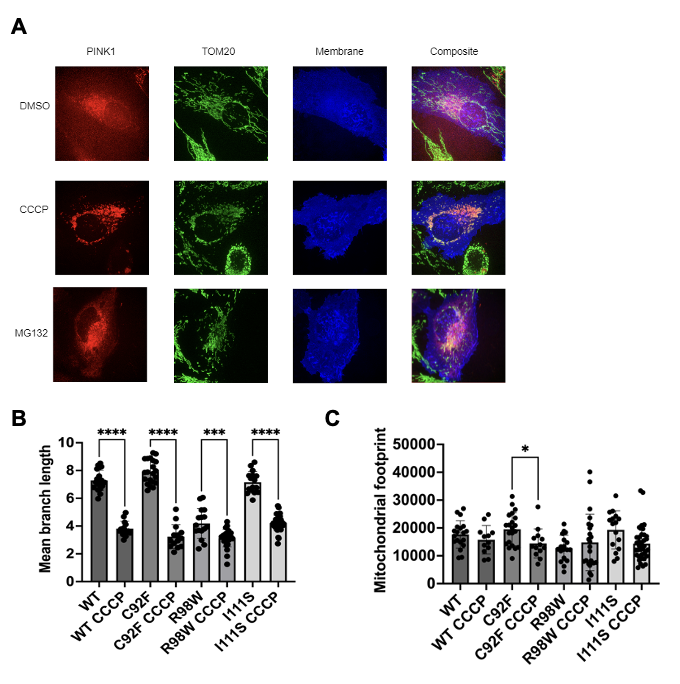


**Supplemental Fig 2. A**. WT-PINK1-mCherry transfected HeLa cells were treated with DMSO, CCCP, and MG132 for 6 hrs. prior to fixing the cells for confocal microscopy. **B** Quantification of mitochondrial mean branch length under DMSO or CCCP conditions. **C.** Quantification of mitochondrial footprint in under DMSO or CCCP conditions. Data is represented as mean ± SEM (* p < 0.05; *** p < 0.0005, ns denotes no significance). n=4

**
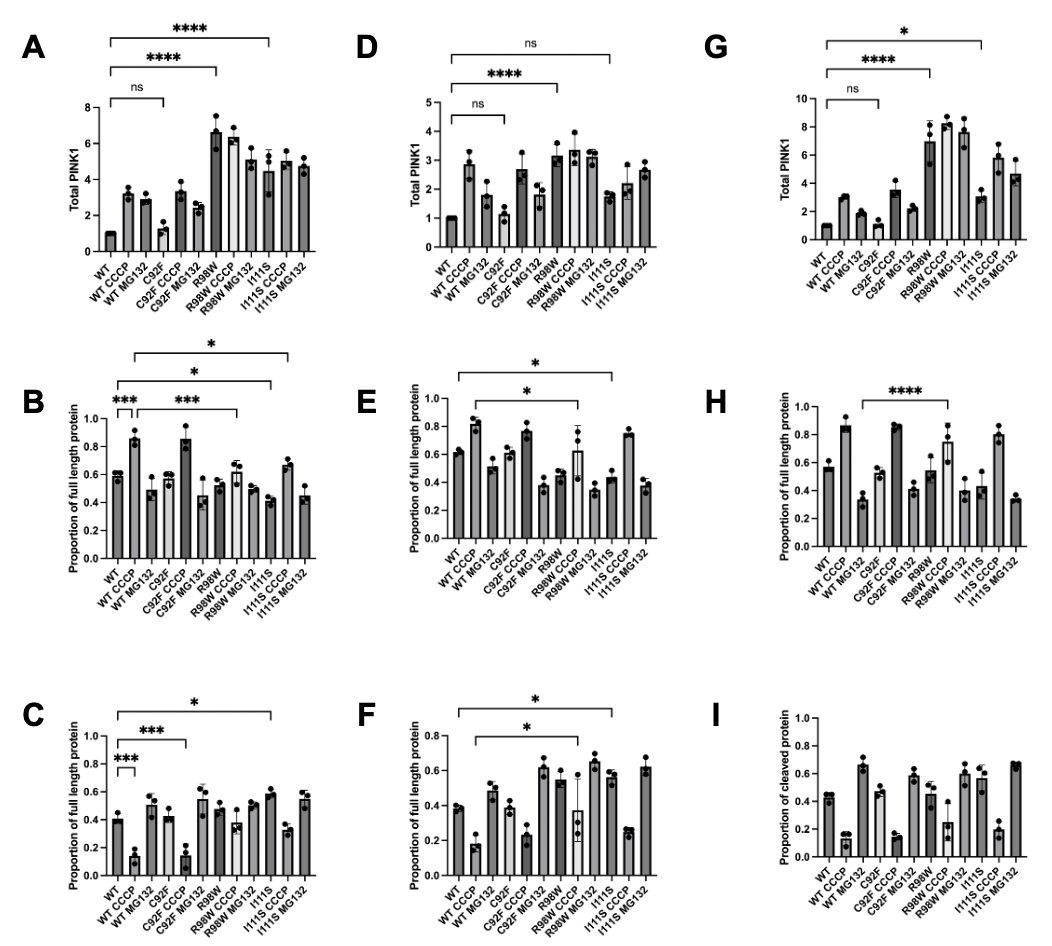
**

**Supplemental Fig. 3. PINK1 variant cleavage in HeLa cell lysates.** Quantification of immunoblots depicting total PINK1-mCherry levels, and proportion of proteolyzed protein in SH-SY5Y (**A,B,C**), bEnd.3 (**D,E,F**) and HeLa **(G,H,I)**. Data is represented as mean ± SEM (* p < 0.05; *** p < 0.0005) n=4.
